# A hybrid controller method with genetic algorithm optimization to measure position and angular for mobile robot motion control

**DOI:** 10.3389/frobt.2022.1087371

**Published:** 2023-01-12

**Authors:** Muhammad Razmi Razali, Ahmad Athif Mohd Faudzi, Abu Ubaidah Shamsudin, Shahrol Mohamaddan

**Affiliations:** ^1^ Faculty of Electrical Engineering, Universiti Teknologi Malaysia, Johor Bahru, Malaysia; ^2^ Centre for Artificial Intelligence and Robotics, Universiti Teknologi Malaysia, Kuala Lumpur, Malaysia; ^3^ Fakulti Kejuruteraan Elektrik dan Elektronik, Universiti Tun Hussein Onn Malaysia, Parit Raja, Malaysia; ^4^ Department of Bioscience and Engineering, College of Systems Engineering and Science, Shibaura Institute of Technology (SIT), Saitama, Japan

**Keywords:** Fuzzy-PID, Pid, GA, Position, Angular

## Abstract

Due to the complexity of autonomous mobile robot’s requirement and drastic technological changes, the safe and efficient path tracking development is becoming complex and requires intensive knowledge and information, thus the demand for advanced algorithm has rapidly increased. Analyzing unstructured gain data has been a growing interest among researchers, resulting in valuable information in many fields such as path planning and motion control. Among those, motion control is a vital part of a fast, secure operation. Yet, current approaches face problems in managing unstructured gain data and producing accurate local planning due to the lack of formulation in the knowledge on the gain optimization. Therefore, this research aims to design a new gain optimization approach to assist researcher in identifying the value of the gain’s product with a qualitative comparative study of the up-to-date controllers. Gains optimization in this context is to classify the near perfect value of the gain’s product and processes. For this, a domain controller will be developed based on the attributes of the Fuzzy-PID parameters. The development of the Fuzzy Logic Controller requires information on the PID controller parameters that will be fuzzified and defuzzied based on the resulting 49 fuzzy rules. Furthermore, this fuzzy inference will be optimized for its usability by a genetic algorithm (GA). It is expected that the domain controller will give a positive impact to the path planning position and angular PID controller algorithm that meet the autonomous demand.

## 1 Introduction

Mobile robotics is a branch of robotics that studies how to make mobile automatic equipment function better in various situations. The mobile robot is capable of determining the shortest, most direct route between its starting and destination points. Obstacle recognition, collision avoidance, and reaching the intended destination are the primary goals of the mobile robot in both familiar and unfamiliar situations. In general, the tasks of a mobile robot that involve human following can be broken down into the following three parts (Hu et al., 2014; Yuan et al., 2018), human detection based on sensors, human motion state/intention estimation and human-following control of the mobile robot. Human detection is the initial step of human following for patient monitoring and rehabilitation. It is vital for safety, the achievement of the robot’s tasks, and natural human-robot interaction because it is the first step in human following. In addition, in order to ensure the safety and effectiveness of the human-following task for patient monitoring and rehabilitation, the mobile robot needs to move as quickly as possible to the goal position and orientation generated based on the human-following rule. This requires strong real-time and accuracy performances from the robot motion control system A variety of controllers and sensors are currently being employed to reduce the bad decisions in object recognition using artificial intelligence, fuzzy logic, and neural networks (Aqeel Ur and Cai 2020). In order to reduce the amount of stopping and achieve the shortest distance between the starting point and the goal places, the specifications of the optimized trajectories should be designed to prevent minor rotation radius and minimize the number of turns (Al-Araji et al., 2018). Since mobile robots are needed for a variety of purposes, including science, education, industry, mining, the entertainment sector, security, military, and search and rescue, their motion control systems must keep on the track of and perform path planning.

As a result of its significance, research into mobile robots is still ongoing (Al-Araji and Yousif 2017). One of the most difficult issues in mobile robot navigation is obstacle recognition. When the obstacles are in motion, the situation becomes more difficult. The real position of dynamic barriers may not be accurately measured in global path planning when the laser sensor is employed to map the environment. Depending on the situation, a local path planning may be able to solve the problem (Hank and Haddad 2016). In addition, design and development of the mobile robot controller is crucial since it is the performance of a mobile robot controller that determines its ability to work (Lee et al., 2018). Mobile robot autonomous navigation is inadequately known as an environment utilizing a hybrid approach. Fuzzy logic controllers (FLCs) have been increasingly popular in recent years due to their flexibility and adaptability. The PID controller is a popular choice among scientists due to its numerous potentials uses and easy deployment in practical use. The most difficult part of using a PID controller is to determine the appropriate gains. Reinforcement learning (RL) is becoming more significant in real control applications due to the benefits of dealing with Riccati equations and Hamilton-Jacobi-Bellman (HJB) equations, which are impossible to solve directly (Dao et al., 2020; Dao and Liu 2021; Vu et al., 2021). Actor/critic structures with Neural Networks (NNs) were presented to construct iterative algorithms with sequential tuning (He et al., 2019), (Luo et al., 2019) to get an approximation of the best control solution (Bhasin et al., 2013).

Different types of controllers have been designed to accomplish a variety of functions, including moving items around, tracking trajectories to monitor the environment, and going on long-term missions or intrusions into potentially dangerous places for people. Research done (Zhao et al., 2019), for the purpose of following the trajectory of autonomous vehicles, a genetic algorithm based on PID controllers has been developed. Both linear and circular trajectories were used in the assessment of the tracking controller that was built for its effectiveness. A project by (Kamil et al., 2019) generally focused on the building of maps for inspection and navigation for and autonomous robot that used LiDAR sensor to provide range between robot and obstacle, the DC motor will drive around the robot and Raspberry-pi will transmit data to PC to perform simultaneous localization and mapping. According to the reviewed literatures, the proportional-integral-differential (PID) controller is the controller that is used the most frequently for path tracking control but it is the controller that is most affected by noise degradation related to derivative control (Haruna et al., 2021). Research done by (Solano et al., 2021), utilizing fuzzy logic to adjust the input from 9 infrared sensors (IR) that are used for environment perceiving in order to increase the resilience and performance of PID controllers. These values are entered into a PID controller, which helps to direct the robot in the appropriate direction at each given angle. The robot has a reactive behavior, which means that it moves around in its environment without a predetermined course.

The use of fuzzy logic to control a single shared controller is becoming increasingly impossible to ignore as designed by (Zahid and Bi 2020). A brain-controlled mobile robot’s safety is ensured by tracking its user’s intents. Recent developments in (Campos et al., 2018) have heightened the need for making use of the Fuzzy PD + I control structure, which is simple in construction and includes a linear velocity controller and an angle fuzzy controller for trajectory control, as well as tuning parameters such as gains at the controller’s input and membership functions through PSO algorithm (form). Research done by (Parikh et al., 2018) proposed on the Fuzzy PID was shown to be more adaptable than conventional PID for the control of a DC motor, according to this study, which compared the performance of both methods. Research done by (Singh and Thongam 2018) presented a mobile robot’s wheels movement at different speeds to avoid a group of clustered obstacles, where the author proposes a fuzzy logic controller to handle navigation in a static environment. However, a major problem with this kind of application is in gain optimization. The objectives of this research are to develop a hybrid controller of Fuzzy-PID optimized by Genetic Algorithm cascaded with 2 DOF classical PID for a mobile robot angular and distance control. Secondly, to control the mobile robot with developed hybrid controller for path planning to obtain targeted angle and distance. Lastly, to validate the performance of the developed controller path planning on a Turtlebot3 and evaluate the consistency of error, $e\left(t\right)$ , delta error, $de\left(t\right)$ , control signal distance, CSD and control signal angle, CSA in gazebo simulator.

The generation of solutions for combined optimization and search issues is a popular application of the GA optimization process, which is commonly employed. This approach adheres to the fundamental elements of genetics and the theory of natural selection. The area of computer science was where the majority of GA’s potential applications were concentrated. On the other hand, strategies based on GA are also utilized within the subject of mobile robot navigation (Hewawasam et al., 2022). The GA begins without any prior knowledge of the optimal solution and is totally dependent on the reactions of both the environment and the evolutionary operators in order to find the optimal solution (Leena and Saju 2014). A FL-based approach was proposed by (Patle et al., 2018) as a means for robots to navigate through unknown dynamic settings. This particular setup made use of a singleton type-1 FL controller in conjunction with a Fuzzy-Wind Driven Optimization (WDO) method. In order to maximize the efficiency of the FL controller’s input and output membership functions, the Fuzzy-WDO algorithm was implemented. In order to understand the core principle of WDO, researchers looked at the mobility behavior of very small air parcels throughout an N-dimensional search region. The primary responsibility of the Type-1 FL controller is to protect the robot from collisions and guide it through situations that are either static or dynamic. The controller receives sensory information as its input and produces two output signals in order to drive the left and right motors of the robot. The distance to the first obstacle, the second obstacle to the left, and the third obstacle to the right are the three sensory data inputs. Through a series of eight fuzzy rules, the inputs are linked to the outputs in a logical fashion.

In this research, the system is built utilizing the Fuzzy-PID controller to regulate two inputs which are error, $e\left(t\right)$ and delta error, $de\left(t\right)$ and self-tuning the gains parameters to control the robot’s position and angular. A Fuzzy-PID controller strategy comprised 7 Membership Rules using Mamdani Fuzzy Rule-Base System. Both position and angular PID controller gains strategy will have constant value throughout the simulation. The development of the Fuzzy Logic Controller (FLC) requires information on the PID controller parameters will be fuzzified and defuzzied based on the resulting 49 fuzzy rules and centroid method. Furthermore, these fuzzy gains will be optimized for its usability by genetic algorithm (GA). It is expected that the domain controller will give a positive impact to the path planning position and angular PID controller algorithm that meet the autonomous demand.

This paper has been divided into four parts. Section II deals with Fuzz-PID gain tuning technique and parameters. Section III will discuss the Genetic Algorithm (GA) optimization technique. Section IV will discuss the path planning algorithm. Next, Section V describes the simulation results of the performance of the path planning algorithm with PID controller in angle and coordinates measurements. Finally, Section VI concludes the paper with future studies and conclusions.

## 2 Fuzzy system for tuning the PID gains

### 2.1 PID controller system

The Proportional Integral Derivative (PID) controller and a fuzzy system for fine-tuning the PID gains will be discussed in this section for stabilizing the Turtlebot3. Turtlebot3 is a small, affordable, programmable, ROS-based mobile robot for use in education, research, hobby, and product prototyping.

Mobile robots frequently use PID controllers for feedback. An input value is calculated by subtracting the collected data from a reference value and using this difference to determine how close the system data should come to or stay at the reference value (Jamshidi et al., 2018). The PID controller may regulate the input value based on previous data and the different appearance rate, resulting in a more accurate and stable system that is easier to maintain. Derivative controllers comprise the proportional controller and the integrated controller. For example, a gain amplifier like a proportional controller can be used. Note that system stability will be decreased as a result of the reduction in steady-state error. Errors at a steady state can be eliminated by using an integrated controller. The response time of the system can be sped up by using a derivate controller (Chang and Chang 2019).

$K_{p}$ , $K_{i}$ and $K_{d}$ are the gains in proportional, integral and derivative form. The PID controller’s mathematical formula is as in Equation 1. The PID controller’s success is dependent on the PID gains being selected correctly. Getting the PID gains just right is no easy process. Experienced human experts are frequently used to fine-tune the PID gains. Fuzzy IF-THEN rules (Mamdani Fuzzy Rule-Base Systems) for the PID gains will be determined in the next step, and then a fuzzy system will be utilized to change the PID gains on-line. The Ziegler-Nichols method will be used to alter controller parameters, and the system’s response will be shown in Equation 2. By starting with a PID controller instead of from scratch, the performance will be fine-tuned to the desired level before adding by the fuzzy logic to it.

$$G_{s}\left(s\right)=K_{p}+\frac{K_{i}}{s}+K_{d}s$$ (1)

$$u\left(t\right)=K_{p}e\left(t\right)+K_{i}\int_{0}^{t}e\left(t\right)dt+K_{d}\frac{de\left(t\right)}{dt}$$ (2)

where $u\left(t\right)$ is the control signal and $e\left(t\right)$ is the error reference and desired position of the robot. Proportional-integral-derivative (PID) controllers are the most often used industrial process control controllers because of their simple construction and reliable performance. Researchers have drawn toward use the traditional PID controller due to the versatility it offers in a variety of contexts. However, the most challenging aspect of PID is determining the appropriate values for the gains (Aqeel Ur and Cai 2020). The design of a fuzzy-PID controller by (Lee et al., 2018) is proposed for use in path tracking by a mobile robot equipped with differential drive. A PID controller plus a fuzzy controller with two inputs and three outputs are the components that make up the fuzzy-PID controller. The fuzzy controller is able to tune the parameters of the PID controller when the system response contains both an error and an error rate.

**FIGURE 1:**
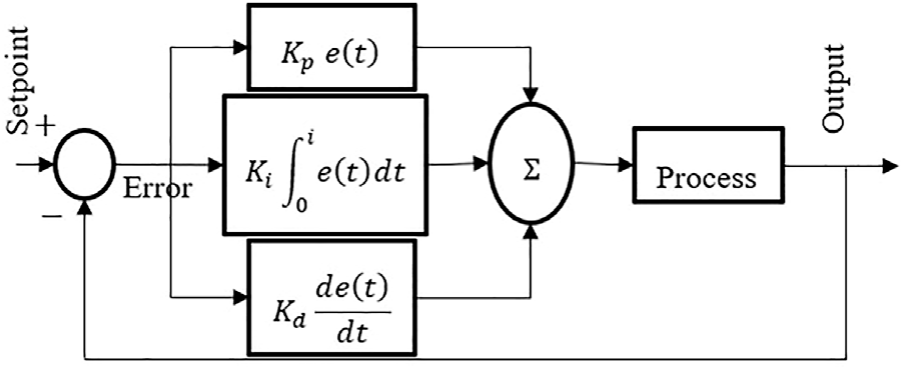
Basic PID controller.

Fuzzification, knowledge base, fuzzy inference, and defuzzification are all necessary steps in the fuzzy logic controller process (Muhammad et al., 2019). There are two inputs that are first fuzzified and then processed by the Fuzzy inference module utilizing heuristic decisions before being sent into the defuzzification module. Defuzzification technique adjusts PID gains and provides the tuned $K_{p}$ , $K_{i}$ and $K_{d}$ output values even when the mobile robot’s dynamics are altered throughout the execution. The common transfer function of PID controller can be expressed as in Figure 1.

### 2.2 Fuzzy controller system

At the moment, the classic PID control method and the fuzzy control method are the most common types of control methods (Lin and Ni 2018). Traditional PID control algorithms are characterized by their straightforward structures and high levels of popularity; non-etheless, an accurate mathematical model is required in order to provide superior linear system control. PID control is restricted for use with control objects that have high hysteresis and inertia as well as complex signals for tracking, as is common knowledge (Babunski et al., 2020). On the other hand, the control effect that it has on non-linear systems is not ideal. It might be challenging to eradicate steady-state mistakes in fuzzy control systems, despite the fact that the fuzzy control method does not need for precise mathematical models (XU et al., 2009).

A fuzzy logic controller, also known as an FLC, has the ability to work with uncertain data and situations. Fuzzy-PID control can be used in this context to allow better control over the unfavorable aspects of PID controllers. Within the scope of this discussion, the conventional PID controller has served as the foundation for the construction of the fuzzy PID controller (Zhou et al., 2019). The fuzzifier, the fuzzification rules, the fuzzy inference system, and the defuzzification process are the components that make up a fuzzy logic system (Pour et al., 2022). In contrast to the conventional way of control, the Fuzzy-PID control method may be applied to the path optimization in a dynamic environment. This method possesses the benefits of flexible fuzzy control and strong adaptability, and it can be used in place of the conventional method of control.

**FIGURE 2:**
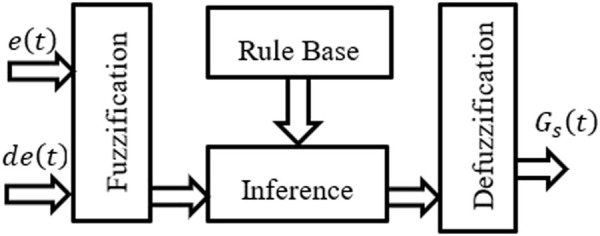
Fuzzy controller internal structure.

The FLC is a machine control method commonly employed. However, FLC has a distinct benefit over genetic algorithms and neural networks: It can be used to solve issues by a person. Because of this, controller design can benefit from their knowledge. This simplifies the management of several machines (Chang and Chang 2019). There are four components to FLC: Fuzzification, Rule Base, Inference, and Defuzzification. The Fuzzy controller’s internal structure as shown in Figure 2.

The conventional PID controller is used as a base for a Fuzzy-PID controller, which regulates PID gains *via* fuzzy reasoning and a changeable discourse Universe. Fuzzy system characteristics such as robustness and adaptability can be used to better tune PID gains in the control approach. The phrase “self-tuning” refers to the ability of the controller to autonomously modify its controlling parameters so that the gains that result in the best possible process output can be achieved. The fuzzy self-tuning PID controller is based on theoretical and practical examination of control rules. In this way, the gains $K_{p}$ , $K_{i}$ and $K_{d}$ can be fine-tuned online in conjunction with other controlling parameters and circumstances. This results in a higher level of overall control precision and thus better performance than a simple fuzzy PID controller with no self-tuning ability, like the PID controller. The Fuzzy controller’s internal structure is depicted as in Figure 2.

Error, $e\left(t\right)$ and rate of change $\frac{de\left(t\right)}{dt}$ are used as inputs to a Fuzzy-PID controller, which uses fuzzy controller rules to adjust PID gains on-line. In the context of PID controller self-tuning, it refers to determining the fuzzy relationship between the three gains of PID ( $K_{p}$ , $K_{i}$ , $K_{d}$ ) and $e\left(t\right)$ and $\frac{de\left(t\right)}{dt}$ and then changing the three gains to fulfil varied requirements for control gains when $e\left(t\right)$ and $\frac{de\left(t\right)}{dt}$ are different. For selecting the language variables of $e\left(t\right)$ and $\frac{de\left(t\right)}{dt}$ , seven membership functions for the error linguistic variable and seven membership function for tits derivative are considered, resulting 49 fuzzy rules. All the rules are detailed in Table 1. The proposed membership functions are formed by triangles and trapezoids with 7 partitions such as: Dismal, Poor, Mediocre, Average, Decent, Good, Excellent and for outputs we have chosen seven fuzzy rules (NB, NM, NS, Z, PS, PM, PB) where NB denotes Negative Big, NM denotes Negative Medium, Negative Small (NS), Zero (Z), Positive Small (PS), Positive Medium (PM) and PB denotes as Positive Big. The inference rules presented in Table 1 can be read as follows: For example, IF the error, $e\left(t\right)$ is Poor AND the Delta Error, $de\left(t\right)$ is Poor THEN output, $G_{s}\left(s\right)$ will be Negative Big (NB).

**TABLE 1:** Fuzzy rules.

	Delta Error, $\frac{de\left(t\right)}{dt}$							
Error, *e*(*t*)		Dismal	Poor	Mediocre	Average	Decent	Good	Excellent
	Dismal	NB	NB	NB	NB	NM	NS	Z
	Poor	NB	NB	NB	NM	NS	Z	PS
	Mediocre	NB	NB	NM	NS	Z	PS	PM
	Average	NB	NM	NS	Z	PS	PM	PB
	Decent	NM	NS	Z	PS	PM	PB	PB
	Good	NS	Z	PS	PM	PB	PB	PB
	Excellent	Z	PS	PM	PB	PB	PB	PB

**FIGURE 3:**
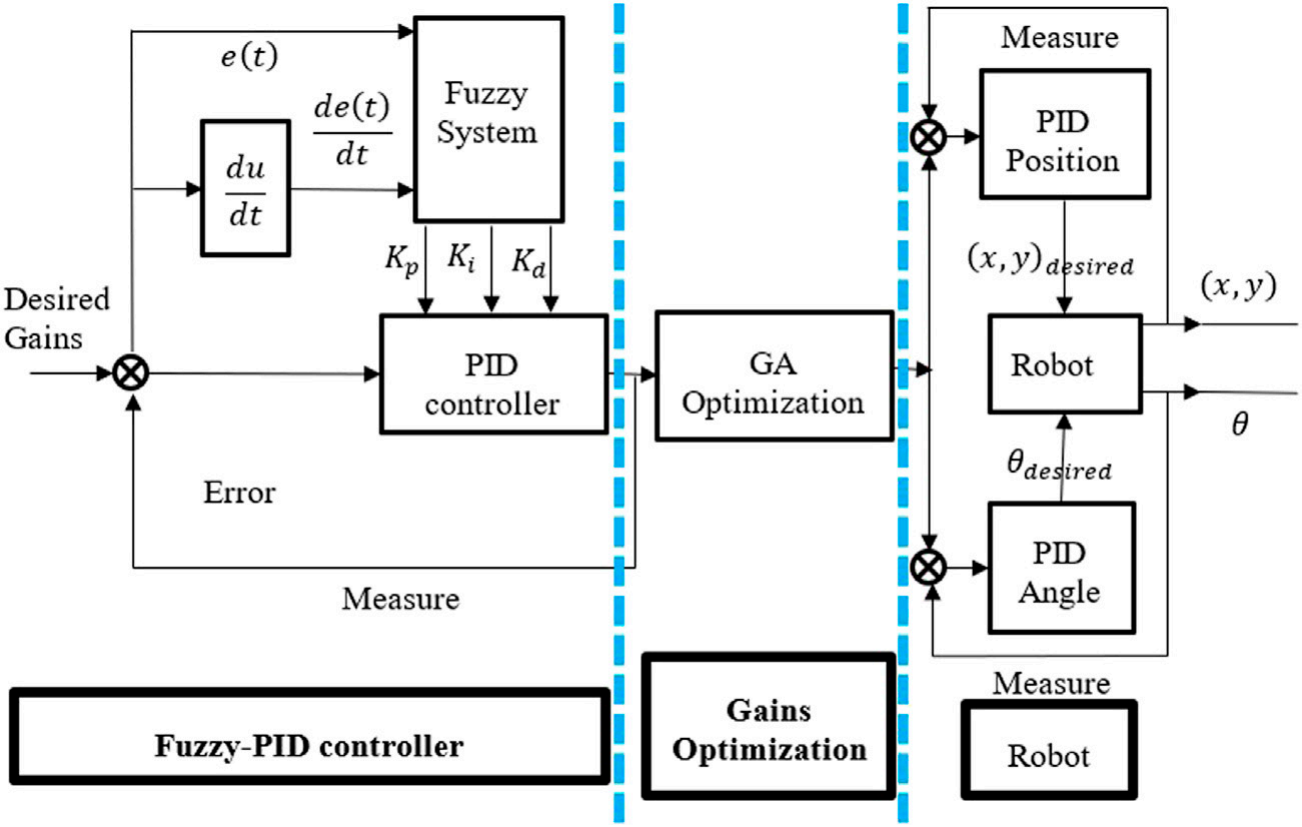
Structure of Turtlebot3 with proposed controller, GA optimization and path planning.

**FIGURE 4:**
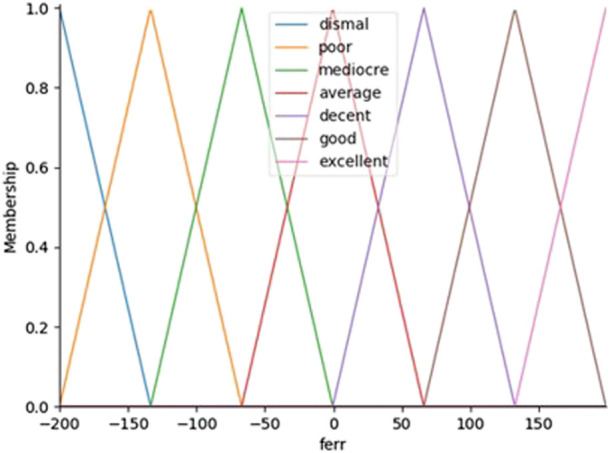
Membership functions vs. the error, $e\left(t\right)$ .

**FIGURE 5:**
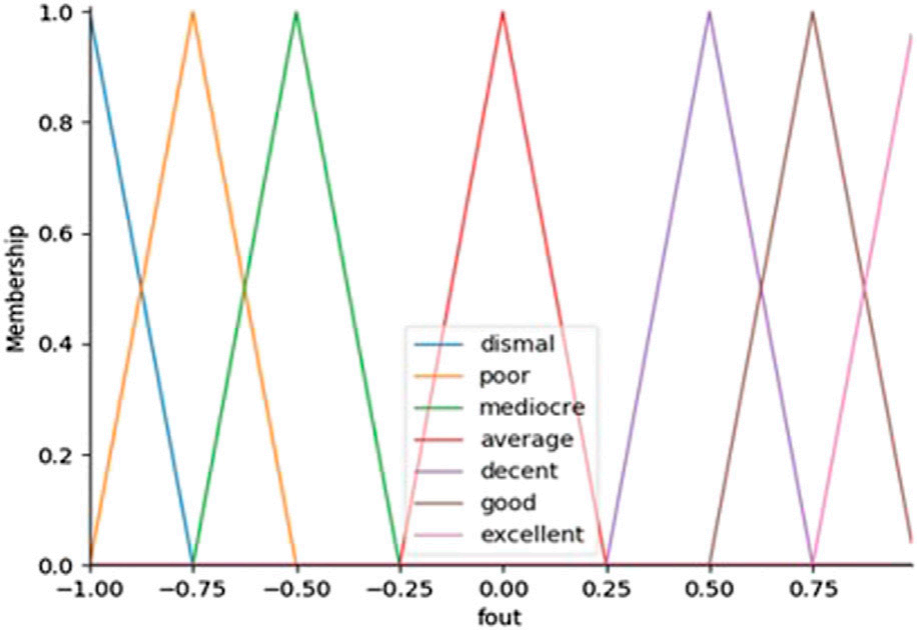
Membership functions vs. the output, $u\left(t\right)$ .

Figure 3 depicts a Fuzzy-PID controller block diagram optimized by the genetic algorithm (GA) cascaded with the two PID controllers. According to the block diagram, the fuzzy system has three outputs $\left(K_{p},\;K_{i},K_{d}\right)$ , which it receives from two inputs. In order to regulate the Turtlebot3 position and angle, in this research two extra controllers, one for each degree of freedom are used. The optimized gains from the Fuzzy-PID controllers are used by the robot to be optimized again by the PID angle and PID position to regulate the path angular and position in the simulation. It has been decided that the membership functions of all the inputs and outputs will be the same. These membership functions are shown in Figure 4 which plotted between membership function and error, $e\left(t\right)$ . Furthermore, Figure 5 shown the membership function plotted between the output, $u\left(t\right)$ .

The antecedent objects hold the Universe variables and membership function will be error rate, $e\left(t\right)$ (ferr) as shown in Figure 1, rate of error, $de\left(t\right)$ (fder) and output, $G_{s}\left(s\right)$ (fout) as shown in Figure 2. The custom membership functions are built interactively with ‘Dismal’, ‘Poor’, ‘Mediocre’, ‘Average’, ‘Decent’, ‘Good’ and ‘Excellent’ and their variables will be [-1, -1, -0.75], [-1, -0.75, -0.5], [-0.75, -0.5, -0.25], [-0.25, 0.0, 0.25], [0.25, 0.5, 0.75], [0.5, 0.75, 1.0], and [0.75, 1.0, 1.0]. In order to compute the high-performance controller, the PID controller that was developed and designed is tuned with fuzzy logics (Wu and Zhang 2011).

The FLC are used to automatically set the gains for PID controllers in SISO plants to achieve a balance between performance and robustness, which in turn yields the PID parameters. The expression for ideal continuous PID controller is shown in Equation 3. After that, the expression for ideal continuous PID controller is shown in Equation 4. After that, these finely tuned Fuzzy outputs are converted into PID controller gains using Equation 5 with the initial value for error, $e\left(t\right)=0$ , delta error, $de\left(t\right)=0$ , $e_{desired}=0$ and $e_{last}=0$

$$e\left(t\right)=e_{desired}-e_{current}$$ (3)

$$de\left(t\right)=e\left(t\right)-e_{last}$$ (4)

$$e_{last}=e\left(t\right)$$ (5)

All inputs and outputs have the same membership functions. The membership functions are consisted of triangular. Controllers employ fuzzy sets with varying widths, which have been found through trial and error. The output $K_{p}$ , $K_{i}$ and $K_{d}$ have been chosen to be [0.2 0.7], [0.001 0.01] and [0.1 0.15] in terms of the fuzzy set width. Inputs' error, $e\left(t\right)$ ranges and error rate, $de\left(t\right)$ ranges have been set to [-1 1] and [-10 10], respectively, and if any inputs fall outside of these parameters, the algorithm employ saturation to bring them back inside the acceptable range.

A fuzzy controller would not be complete without a set of linguistic rules. When an expert’s experience can be easily translated into these rules, the controller’s actions can be defined by an infinite number of such rules. In certain circumstances, these rules are derived by a process of trial and error.

In this research is presented a two-point, three-output system where, $K_{p}$ , $K_{i}$ and $K_{d}$ are the outputs of the fuzzy logic controller, and $e\left(t\right)$ and $\frac{de\left(t\right)}{dt}$ are the inputs. The input data is sent into the fuzzification system, where it is recommended by a fuzzy inference system, and then into defuzzification system, where it is tuned to provide the output values of $K_{p}$ , $K_{i}$ and $K_{d}$ .

**FIGURE 6:**
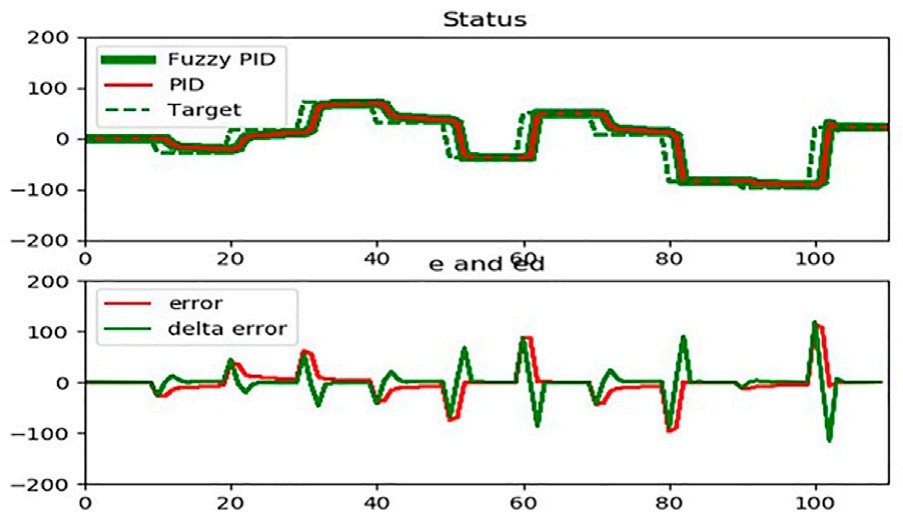
The correlation for error, $e\left(t\right)$ and delta error, $de\left(t\right)$ .

Figure 6 depicts the fuzzy sets used in the membership functions for two input variables (i.e., error, $e\left(t\right)$ , and delta error, $de\left(t\right)$ ) while the membership functions for output variables, $G_{s}\left(s\right)$ , employ fuzzy sets with output values of [-1, 1, 0.01]. Between [-200, 200] and [-200, 200] are the ranges for error, $e\left(t\right)$ and delta error, $de\left(t\right)$ , respectively. In Figure 6, in the Status, dashed line plot is target value, red dashed line is PID and green line is Fuzzy-PID.

### 2.3 Defuzzification method

**FIGURE 7:**
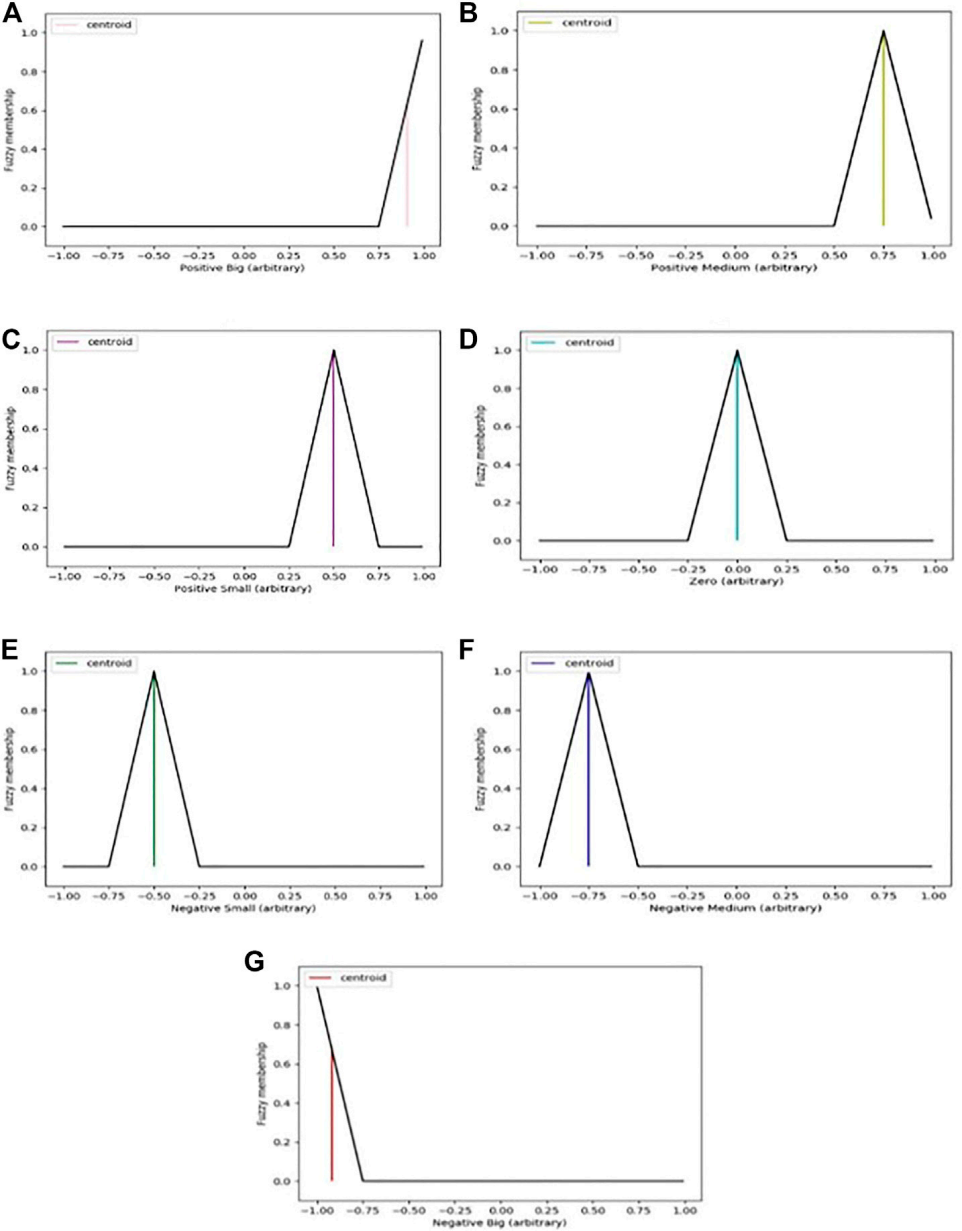
**(A**–**G)**: Arbitrary result for defuzzification using centroid method.

Methods for defuzzification can be categorized into the following four groups: those that provide a real value, those that provide a real interval, those that allow for the ranking of possible distributions, and those that evaluate dispersion (Talon and Curt 2017). The defuzzification technique that was applied to this work is connected to the centroid values of the signal’s global distribution. Due to this fact, the defuzzification techniques that were chosen represent the centroid. Centroid method consists in finding the center of the area under the curve for intervals a and b. This can be expressed as shown in Equation 6:

$$z^{*}=\frac{\overset{b}{\underset{a}{\sum }}u\left(z\right)zdz}{\overset{b}{\underset{a}{\sum }}u\left(z\right)dz}$$ (6)

where $z^{*}$ represents the crips output, $u\left(z\right)$ corresponds to membership function and z is the output variable. For this research, the centroid defuzzification method were implemented. In fuzzy control systems, the output from the systems often consists of a number of different control parameters (Chen et al., 2022). Before the fuzzy outputs may be applied in control systems, defuzzification must be performed on them. In Figure 7 (a) to (g) shown in this section, the determination of defuzzification for the proposed Fuzzy-PID controller is visualized.

The center of gravity method was selected as the defuzzification technique for the controller because of its ease of use, precision, and dependability in addressing the appropriate crisp value for any given fuzzy outputs.

## 3 Genetic algorithm (GA) optimization

An optimization algorithm that is based on the mechanism of biological evolution is known as a genetic algorithm. This algorithm is typically broken down into several steps, including the generation of an initial population, the application of genetic operators for genetic operation, the determination of the fitness value of individuals, and iteratively obtaining the best individuals possible (Li et al., 2020). The genetic algorithm is a method for optimization that simulates the process of evolution as it occurs in biological systems by repeatedly subjecting solutions to testing. When trying to solve an issue involving minimization, it is unrealistic to undertake a comprehensive search because it takes a significant amount of time to provide the best gain. Therefore, in order to cut down on the amount of time required for computing, a local optimal solution rather than a global optimal solution is sought after (Nonoyama and Nishi 2021).

The genetic algorithm for determining the gain can be seen in the following example. By carrying out the process a set number of times, the objective is to achieve the desired result of developing a better solution. The algorithm is run five times, and out of all the solutions that are obtained, the one with the best gain value is the one that is output as the solution. Because the answer is determined by the random numbers that are created, it is necessary to run the method more than once because there is a possibility that it will fall in a bad local solution that will not be suitable for several trials.

The decision variables: distance gains $K_{p_{distance}}$ , $K_{i_{distance}}$ , $K_{d_{distance}}$ , angular gains $K_{p_{angle}}$ , $K_{i_{angle}}$ , $K_{d_{angle}}$ are initialized by the generated gains from Fuzzy-PID controller in the range [-1 1]. This set is considered as one solution and this is repeated 110 times and the collection of 8 solutions is considered as a generation, which is considered as the first generation. In this research used the decimal representations for generation, one point crossover and uniform mutation.

### 3.1 Equation implementation

The model starts by presenting the equation that this research is going to implement. The equation is shown in Equation 7:

$$Y=w_{1}x_{1}+w_{2}x_{2}+w_{3}x_{3}+w_{4}x_{4}+w_{5}x_{5}+w_{6}x_{6}$$ (7)

The equation has 6 inputs ( $x_{1}$ to $x_{6}$ ) and 6 weights ( $w_{1}$ to $w_{6}$ ) as shown and inputs values are $\left(x_{1},x_{2},x_{3},x_{4},x_{5},x_{6}\right)$ = $\left(K_{p_{distance}},K_{i_{distance}},K_{d_{distance}},K_{p_{angle}},K_{i_{angle}},\;K_{d_{angle}}\right)$ . In this line of research, the goal is to identify the parameters (weights) that will yield the highest possible value for such an equation. The concept of optimizing such an equation appears to be straightforward. The positive input is going to be multiplied by the largest positive number that is even remotely possible, and the negative number is going to be multiplied by the smallest negative number that is even remotely possible. However, the goal of this research is to figure out how to have GA accomplish that on its own so that it can determine that it is preferable to use positive weights with positive inputs and negative weights with negative inputs. This is the idea that will be implemented. The following step is to specify the initializing population size. Because there are so many weights, there will undoubtedly be a total of six generations of each solution in the population. One generation will represent each weight.

### 3.2 Population initialization

The quality of the result is determined by the quality of the primary population that is used in a genetic algorithm. In the context of this study, the Fuzzy-PID controller serves as the heuristic initialization information for path planning. The goal is to increase the gains value and achieve more stability. At this point, the algorithms are in a position to generate the initial population in a random way. It will take on a form that corresponds to the parameters that were chosen (8,6). There are 8 solutions and each one has 6 generations, one for each weight. The population is as follows in Table 2:

**TABLE 2:** Generated population randomly.

0.04353793	0.01146844	0.05452081	0.00707252	0.05893077	0.03578277
0.01024827	0.02711178	0.05636492	0.04308384	0.05548589	0.01598261
0.01775971	0.0502044	0.05764063	0.0031841	0.0083322	0.021976
0.0565583	0.00390275	0.03074476	0.04099405	0.04241032	0.00698859
0.03449538	0.05483135	0.05782144	0.00149375	0.04184159	0.04018424
0.010176	0.02429801	0.03079434	0.04368901	0.01202527	0.04048726
0.05691229	0.0072845	0.00875476	0.05215794	0.03623459	0.01189234
0.03303685	0.02941146	0.01406343	0.03646531	0.03760042	0.0197072

Given that it is generated in a random way, it will undoubtedly be different when it is run once again. In the generational model, this research produces ‘n’ offspring, where n is the population size; at the end of each iteration, the entire population is replaced by the new population, therefore n represents the number of offspring produced.

### 3.3 Fitness function

After the population has been prepared, the algorithms will begin the process of selecting the optimal solution from the existing population by making use of the fitness function. The fitness function is able to accurately analyze the advantages and disadvantages of each gain, which is often proportional to the fitness value, and it has a significant impact on the genetic algorithm’s capacity to converge on optimal solutions and remain stable over time. The goal of this research is to improve the stability of the Turtlebot3 motion so that it can move further without becoming unstable, as well as to make the Turtlebot3 motion operate more smoothly. As a result, the values of the gains and the degree to which the increases are coherent have been optimized. The fitness function can be defined in its most basic form as a function that takes a candidate solution to the problem as its input and produces, as its output, a measure of how “fit” or “good” the answer is in relation to the problem that is being considered. Since a GA requires constant calculation of the fitness value, the algorithm used for this purpose needs to be as efficient as possible. A selection of eight solutions at random is made from the current generation, and the evaluation values of those answers are utilized to choose the individuals who will be carried forward to the next-generation. The fitness function is designed as follows:

$$Fitness=\sum \left(population\times Y\right)$$ (8)

The fitness function accepts both the equation inputs values ( $x_{1}$ to $x_{6}$ ) in addition to the population. According to the equation, the fitness value is determined by taking the sum of the products (SOP) that are found between each input and the weight according to Equation 8. There will be a certain number of SOPs that correspond to the total number of solutions that can be applied to each population. Due to the fact that the number of solutions was previously determined to be eight, there will be eight SOPs as stated below. Take note that the solution is in significantly better shape the higher the fitness value.

### 3.4 Crossover operator

The next-generation is split into two groups, with one individual coming from each group. Next, a random number between 0 and 0.06 is created, and the crossover process is carried out if that number is lower than the rate at which it is expected to occur. After the selection operation is complete in a genetic algorithm, the next step is the crossover operation, which is the fundamental process of generation rearrangement. When the genetic operation is successful, the choice of the intersection is determined by random; hence, either the single-point crossover or the multi-point crossing may be implemented. The single-point crossover approach will be utilized in this research study. Finding all of the same points in the two groups is the first step of the specific crossover operation. After that, one of the groups at random will be choose to operate on, and then finally cross the following paths.

### 3.5 Mutation operator

A random number in the range of [0 0.06] is generated for each solution in the next-generation, and when the mutation rate is less than the set mutation rate, mutation is performed. When a mutation occurs the Fuzzy-PID gains value in the range [−1, 1] are generated and input to $K_{p_{distance}}$ , $K_{i_{distance}}$ , $K_{d_{distance}}$ , $K_{p_{angle}}$ , $K_{i_{angle}}$ and $K_{d_{angle}}$ in that solution. However, if the number of generations exceeds 1, random numbers are generated in the range [(optimal solution)-1, (optimal solution) +1] with values close to the optimal solution at that time. Choose a new gains value from the Fuzzy-PID controller instead of the previous gains value randomly. This was how classic genetic algorithms handled random mutation.

### 3.6 Update generation

Keep the population of the following generation at the same level as the population of the generation that is currently in effect. The final stage is to repeat steps 2 through 6 for a total of 5 generations. Within the context of this GA, the initial solution is derived from a series of random numbers. As a result, the quality of the solution does not depend in any way on the configuration of the initial solutions. The final stage of generation as shown in Table 3:

**TABLE 3:** Final stage of generic algorithm generation.

Generation			Best result		
0			2.577973866865198 × 10^−5^		
1			2.6837838542276733 × 10^−5^		
2			2.6837838542276733 × 10^−5^		
3			2.9103608789928096 × 10^−5^		
4			3.1847903853924453 × 10^−5^		
Best Solution					
0.05691229	0.0072045	0.00875476	0.05215794	0.03623459	1.32506896
Best Solution Fitness					
3.184479039 × 10^−5^					
$K_{p_{distance}}$			0.05691229		
$K_{i_{distance}}$			0.0072045		
$K_{d_{distance}}$			0.00875476		
$K_{p_{angle}}$			0.05215794		
$K_{i_{angle}}$			0.03623459		
$K_{d_{angle}}$			1.32506896		

## 4 Path planning algorithm

In this section, this paper will describe the design and implementation of the path planning algorithm. The main objective of this algorithm is to move the mobile robot to the target position from an initial coordinate to another final one. The fuzzy tuned PID controller is provided with coordinate information and mobile robot angles, and tracks the output intention of Fuzzy tuned PID controller. The robot path planning algorithm is based on the shared control compromises of a direct control mode and autonomous control mode. The shared controller sends steering commands (i.e., position and angular) to the robot, taking the path tracking situation into account, rather than directly executing the user’s commands.

In Figure 3 it is shown that the structure of Turtlebot3 with Fuzzy-PID controller cascaded with 2 degree of freedom (DOF) PID controller block diagram. According to the block diagram, the fuzzy system has three outputs ( $K_{p}$ , $K_{i}$ , $K_{d}$ ), received from two inputs which are error, $e\left(t\right)$ and delta error, $\frac{de\left(t\right)}{dt}$ . In order to regulate the Turtlebot3 position and angle, this research will use two extra controllers, one for each degree of freedom shown in the block diagram.

### 4.1 Position and angular PID algorithm

**FIGURE 8:**
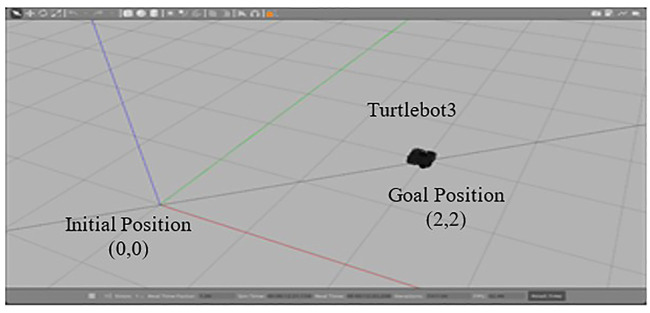
Gazebo Simulator with Turtlebot3 environment.

During this study, two types of path planning algorithms were developed in order to collect input from the Fuzzy tuned PID controller and then plan the reference path to the mobile robot from the beginning point to the goal position in the environment with collision-free navigation. The gazebo simulator environment and a kinematic model of the mobile robot of Turtblebot3 are utilized as platforms for the mobile robot to move in a constrained two-dimensional $\left(x,y\right)$ range from the initial position to the goal position in order to study these methods. The mobile robot model and the simulator environment are needed as a platform for the position and angular position algorithm as shown in Figure 8.

For the purpose of determining the robot’s position, Trigonometry and Pythagoras’s theorem are used to measure the robot’s position between two centers. Equation 9 will be used to find the Turtlebot3’s goal position from the initial position. Then, for the estimation of angular position of the robot, the arctangent function has been used to determine the angular position as shown in Equation 11.

$$Distance=\sqrt{{\left(x_{goal}-x_{initial}\right)}^{2}+{\left(y_{goal}-y_{initial}\right)}^{2}}$$ (9)

Difference Distance=Distance−Previous DistancePrevious Distance=0 (10)

$$Path\;Angle=\mathrm{atan}2\left(y_{goal}-y_{initial},x_{goal}-x_{initial}\right)$$ (11)

An algorithm is devised with the combination of the PID controller and the resulting Equation 9 to find the robot movement processes to see if the two methods gave the correct measurement in data plotting to understand how the Control Signal Distance, CSD regulates between initial and goal positions as in resulting Equation 12.

$$CSD=\left(K_{p_{distance}}\times error\;distance\right)+\left(integral\;error\;distance+\left(K_{i_{distance}}\times integral\;error\right)\right)+\left(K_{d_{dsitance}}\times \left(\frac{error\;distance-last\;error\;distance}{delta\;time}\right)\right)$$ (12)

error distance=distance−0.01 (13)

$$integral\;error\;distance=\left(error\;distance\times delta\;time\right)$$ (14)

last error distance=error distance (15)

An algorithm is devised with the combination of the PID controller and the resulting Equation 4 to find the robot movement processes to see if the two methods gave the correct measurement in data plotting to understand how the Control Signal Distance, CSD regulates between initial and goal positions as in Equation 16.

$$CSA=\left(K_{p_{angle}}\times error\;angle\right)+\left(integral\;error+\left(K_{i_{angle}}\times integral\;error\;angle\right)\right)+\left(K_{d_{angle}}\times \left(\frac{error\;angle-last\;error\;angle}{delta\;time}\right)\right)$$ (16)

$$error\;angle=path\;angle+\frac{\pi }{2}+2.5$$ (17)

$$integral\;error\;angle=\left(error\;angle\times delta\;time\right)$$ (18)

last error angle=error angle (19)

## 5 Simulation results and discussion

In order to test the performance of proposed Fuzzy-based assistive controller developed in Section II, this study used gazebo simulator in Robotic Operating System (ROS). In this research, the performance of the Fuzzy-Tuned PID controller simulated by a mobile robot has been tested given the specified task. The task completion will be measured by the CSD and CSA result. Eventually, the gains from Ziegler-Nichols PID controller are tuned by the Fuzzy-PID controller ( $K_{p}$ , $K_{i}$ , $K_{d}$ ) values are set at 1.4, 0.04, and 0.03. The initial point of the robot is at (0,0) and the goal point is at (2,2). The desired gains that are proposed in this system is 0.06, 0.03, 0.05 for distance algorithm and 0.06, 0.03, 0.05 for angle algorithm.

### 5.1 Control signal distance, CSD

**FIGURE 9:**
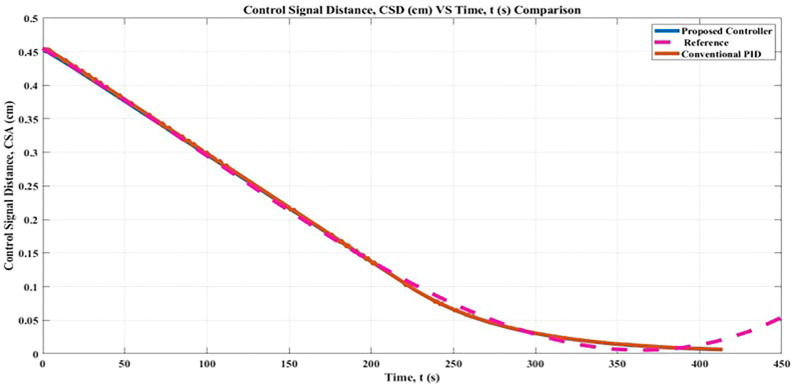
Control Signal Distance (CSD) comparison result.

As shown in Figure 9, the Fuzzy-PID cascaded with 2 PID controller with genetic algorithm (GA) method, which was evaluated by testing the determined controlled distance, achieved the goal of movement from the starting point to the goal point both with and without the proposed controller. This can be seen by comparing the controlled distance with and without the proposed controller. For the purpose of this simulation, the PID gains of the path planning have been held constant, and the distance control has been adjusted so that the result may evaluate how well the FLC works to prevent the robot from swerving off course. As a result, it is plausible to hypothesize that the larger the distance regulated, the greater the likelihood that the CSD will have a small value of Root Mean Square Error (RMSE). According to this distance controlled, the robot moves with regulated uniform performance and bends toward the position it has to be in to achieve the goal.

A favorable outcome can be attributed to the optimal gains obtained in both the Fuzzy-PID hybrid controller and the two PID controllers for the robot position. The conclusion that can be drawn from the data presented in Figure 9 is that the output of CSD with the proposed controller and conventional PID controller prevents the robot movement from deviating from its goal distance, which is zero. The study known as Root Mean Square Error (RMSE) shows that the CSD result with the proposed controller is 0.14 cm closer to the target than it would be without it, which would be 0.15 cm correspondingly. During the simulation in the gazebo, the robot moved with less jerkiness and remained in the ready state it had been programmed to be in. Using the controller that has been proposed, it is possible for the FLC Membership Function to explain the observed controlled distance in CSD. In addition, a Fuzzy-PID controller was implemented, and the FLC learnt how to use it, in order to achieve consistently accurate distance proximity for the robot.

### 5.2 Control signal angle, CSA

The objective of the simulation was to move the simulated mobile robot from the beginning point to the goal point in the least amount of time feasible while avoiding any obstacles in an environment that did not contain any collisions. For CSA, one may conceivably hypothesize that the likelihood of CSA having a high value of Root Mean Square Error (RMSE) increases in proportion to the size of the regulated angle. The lower range for generating angle with regard to *x*-axis for the initial point is 0°and the upper range for generating angle with regard to *x*-axis for the initial point is 0°. These considerations may account for the relatively uniform correlation between the upper and lower range angles, as well as the robot’s final goal point posture.

**FIGURE 10:**
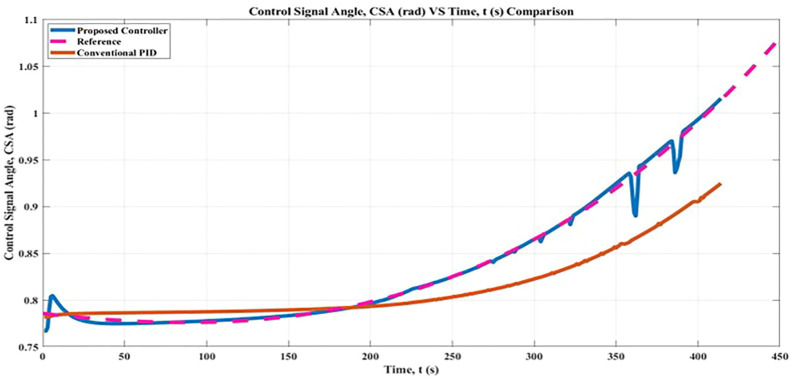
Control Signal Angle, CSA comparison result.

The findings of this study can be interpreted in a number of different ways, as demonstrated in Figure 10, which shows that the results obtained with and without the suggested controller are essentially equivalent. According to the findings of the Root Mean Square Error (RMSE) analysis, the proposed controller leads to a greater proportion of the angle being regulated even while the robot is in motion. The calculated CSA result with the proposed controller was 6.79% better in angle controlled, whereas the result with the conventional PID controller was 3.68%. In addition, it is hard to exclude the potential that the proposed controllers would interfere with the implementation of the angular algorithm. Within the framework of the proposed controller, the fuzzy logic controller for the regulated angle is built through a process of learning and trial within the controller. It is possible to hypothesize that this condition is more likely to occur when there exists a uniform initial angle, $\theta_{initial}$ , and final angle, $\theta_{final}$ . Hence, it could conceivably be hypothesized that the bigger the angle controlled the more likely CSA to have higher RMSE value.

### 5.3 Linear Velocity

**FIGURE 11:**
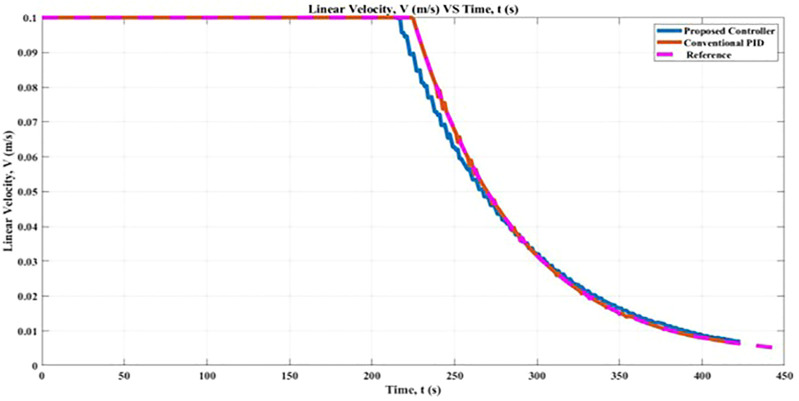
Linear Velocity, V comparison between proposed controller and conventional PID controller.

First, the parameter gains ( $K_{p}$ , $K_{i}$ , $K_{d}$ ) for conventional PID controller are adjusted and it was found that the ideal gains are $K_{p_{PID}}=0.06$ , $K_{i_{PID}}=0.03$ and $K_{d_{PID}}=0.01$ and the ideal gain parameter for proposed controller with $K_{p}=1.4$ , $K_{i}=0.04$ , $K_{d}=0.03$ gains parameters. According to Figure 11, when moving from its starting point (0,0) to its final position, (2,2) the robot moved 4.98% with a satisfactory linear velocity that was set at 0.1 m/s by employing the proposed controller. However, when a conventional PID controller was used, the robot moved at a rate that was 4.99%, faster than the velocity that had been set. The linear velocity of the robot for both controllers can be seen by using RMSE percentage. The conclusion that can be drawn from the findings of this linear velocity experiment is that the robot needs to have a sufficient velocity in order to move with the appropriate controller in the simulation.

In general, therefore, it seems that with the proposed controller resulted in the R-squared value at 0.9979 which performs well compared with the conventional PID which resulted in a value of 0.9974. Based on the Linear Correlation Coefficient method, the strength of the linear relationship between the two variables, the R-squared value for the proposed controller is positive and close to 1, which indicates that it has a strong positive correlation. In the course of this study, the Turtlebot3 robot first makes use of the proposed controller to figure out the optimal linear velocity along the *x*-axis. After that, a genetic algorithm, also known as GA, was introduced to the proposed controller in order to obtain a result that was more precise and accurate. In conclusion, the performance of the proposed controllers in conjunction with GA will be compared to that of a conventional PID controller.

### 5.4 Angular Velocity

**FIGURE 12:**
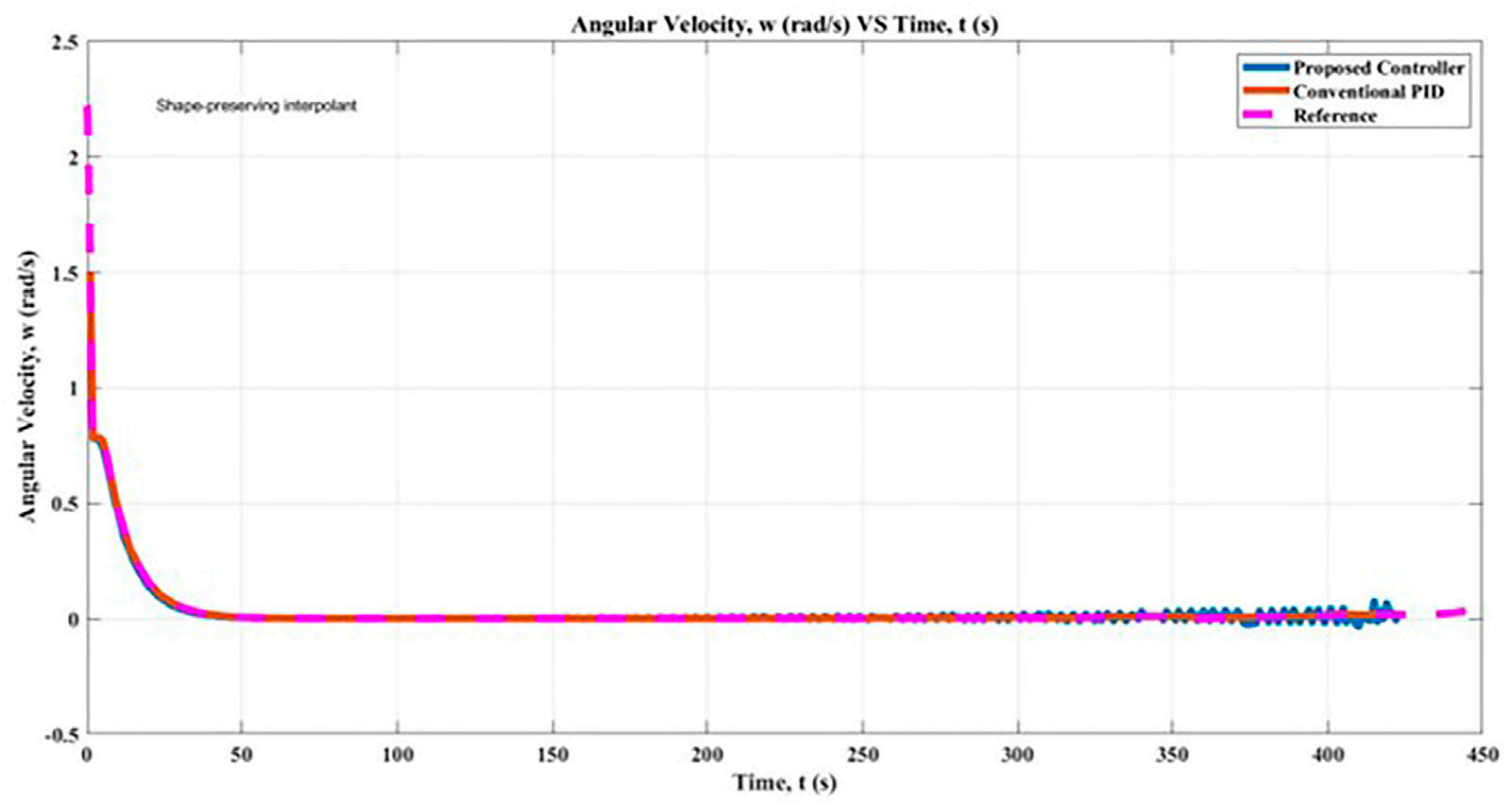
Angular Velocity, α comparison between proposed controller and conventional PID controller.

Figure 12 illustrates a comparison of the simulation that made use of this research by the angular velocity of the robot. The application of the proposed controller results in a marginal increase in the angular velocity of the robot, as determined by the fuzzy rules presented in Table 2 and the findings of the simulation. When the conventional PID controller was used, it recorded an angular velocity that was 98.38% lower than the proposed controller, which resulted in an 98.34% improvement in the robot’s angular velocity while it was in motion. The acceptable angular velocity is defined as 1.5 rad/s. As a result of this research, the controller that was suggested resulted in an improvement in the mobility of the robot towards its goal position in the simulation when comparing its angular velocity. The Fuzzy-PID method, the cascaded PIDs controller, and the GA algorithm had a high association with one another. The findings demonstrated that the online tuning method has the potential to maximize the percentage of the robot’s angular velocity that corresponds to stability and adaptability of motion in the direction of the desired position.

## 6 Conclusion

The goal of this research was to demonstrate a correlation between the Fuzzy-PID controller which its gains optimized by genetic algorithm (GA) and the 2-DOF PID controller, also known as the Position and Angular algorithm method. This was accomplished by comparing the two types of controllers which are with the conventional PID controller. One of the most important things that came out of this research was the finding that the gains tuned by the combination of a fuzzy controller and a PID controller could achieve more precise values. These values could then be used by a mobile robot to carry out a movement that was more effective on its way from the initial point to the goal point. The significance of the improvements is backed up by the data in a clear and convincing way. The second significant finding was that the CSD and CSA have increased the control performance and efficiency of the proposed controllers to the robot movement. The findings of this study provide credence to the hypothesis that, as the robot progresses from its initial position to its goal position, it will be able to successfully implement both algorithms if a fine-tuned PID controller is applied to it. The third notable finding was that the linear velocity and angular velocity of the robot has strengthened the robot’s stability and adaptation of the suggested controller to the robot movement in the simulation. The findings of this study provide evidence to support the hypothesis that, as the robot moves from its starting position to its goal position, it will be able to successfully implement the proposed controller if the simulation is run without any collisions. This hypothesis was tested by running the robot in a collision-free environment.

## Data Availability

The original contributions presented in the study are included in the article/supplementary material, further inquiries can be directed to the corresponding author.
